# Fine particulate matter and polycystic ovarian morphology

**DOI:** 10.1186/s12940-022-00835-1

**Published:** 2022-02-18

**Authors:** Victoria Fruh, Jay Jojo Cheng, Ann Aschengrau, Shruthi Mahalingaiah, Kevin J. Lane

**Affiliations:** 1 Department of Environmental Health, Harvard T.H. Chan School of Public Health, Boston, MA USA; 2 Department of Biostatistics and Medical Informatics, University of Wisconsin, 702 West Johnson Street, Madison, WI USA; 3 Department of Epidemiology, Boston University School of Public Health, Boston, MA USA; 4 Obstetrics and Gynecology, Massachusetts General Hospital, 55 Fruit Street, Boston, MA 02114-2696 USA; 5 Department of Environmental Health, Boston University School of Public Health, Boston, MA USA

**Keywords:** Polycystic ovary morphology (PCOM), Air pollution, Fine particulate matter, Electronic medical records

## Abstract

**Background:**

Polycystic ovary morphology (PCOM) is an ultrasonographic finding that can be present in women with ovulatory disorder and oligomenorrhea due to hypothalamic, pituitary, and ovarian dysfunction. While air pollution has emerged as a possible disrupter of hormone homeostasis, limited research has been conducted on the association between air pollution and PCOM.

**Methods:**

We conducted a longitudinal cohort study using electronic medical records data of 5,492 women with normal ovaries at the first ultrasound that underwent a repeated pelvic ultrasound examination during the study period (2004–2016) at Boston Medical Center. Machine learning text algorithms classified PCOM by ultrasound. We used geocoded home address to determine the ambient annual average PM_2.5_ exposures and categorized into tertiles of exposure. We used Cox Proportional Hazards models on complete data (*n* = 3,994), adjusting for covariates, and additionally stratified by race/ethnicity and body mass index (BMI).

**Results:**

Cumulative exposure to PM_2.5_ during the study ranged from 4.9 to 17.5 µg/m^3^ (mean = 10.0 μg/m^3^). On average, women were 31 years old and 58% were Black/African American. Hazard ratios and 95% confidence intervals (CI) comparing the second and third PM_2.5_ exposure tertile vs. the reference tertile were 1.12 (0.88, 1.43) and 0.89 (0.62, 1.28), respectively. No appreciable differences were observed across race/ethnicity. Among women with BMI ≥ 30 kg/m^2^, we observed weak inverse associations with PCOM for the second (HR: 0.93, 95% CI: 0.66, 1.33) and third tertiles (HR: 0.89, 95% CI: 0.50, 1.57).

**Conclusions:**

In this study of reproductive-aged women, we observed little association between PM_2.5_ concentrations and PCOM incidence. No dose response relationships were observed nor were estimates appreciably different across race/ethnicity within this clinically sourced cohort.

**Supplementary Information:**

The online version contains supplementary material available at 10.1186/s12940-022-00835-1.

## Background

Polycystic ovary morphology (PCOM) is an ultrasonographic finding that can be present in women with ovulatory disorder and oligomenorrhea due to hypothalamic, pituitary, and ovarian dysfunction [1–3]. PCOM may be seen in multiple endocrine states where follicular development is altered, resulting in arrested antral follicles [4]. PCOM has been observed in about 30–50% of patients with functional hypothalamic amenorrhea [5–7] and is more common in women with Cushing’s disease [8]. Women with polycystic ovary syndrome (PCOS), a disease notable for oligomenorrhea and androgen excess, and PCOM have demonstrated higher risks of insulin resistance, dyslipidemia and cardiovascular diseases compared to women with only PCOS [9]. The clinical significance of PCOM alone is undefined as previous literature on direct health impacts of PCOM remains sparse. However, some studies have observed associations between PCOM and elevated anti-Müllerian hormone (AMH) among healthy girls with regular menses [10], as well as a higher severity of primary dysmenorrhea [11].

Air pollution has emerged as a possible disrupter of hormone homeostasis interfering with the female reproductive system [12, 13]. Epidemiologic studies have begun to evaluate specific reproductive health outcomes in relation to environmental air pollution including infertility [14–17], hormone function [18, 19], and menstrual cycle status [20–22]. Likewise, animal studies have investigated associations between ovarian function and air pollution, finding significant decreases in the area occupied by primordial follicles for mice exposed to pollutants from diesel exhaust [23] and changes in AMH levels for mice exposed to fine particulate matter (PM_2.5_) [24]. However, there is a dearth of research on the association between PM_2.5_ and PCOM. One study to date has evaluated PM_2.5_ and PCOS, rather than PCOM [25], and observed an increased risk of PCOS with higher levels of PM_2.5_ [25]. However, this study assessed air pollution one year before diagnosis without investigating potentially longer windows of exposure and diagnosed PCOS via ICD-9-CM codes [25].

In the current study, we investigated the association between PM_2.5_ and PCOM in a population of reproductive-age women receiving clinical care. Women in our study had a minimum of four years of exposure data prior to diagnosis. We hypothesized that higher levels of PM_2.5_ would be associated with increased incidence of PCOM.

## Methods

### Study population

This study was conducted at Boston University Medical Campus (BUMC), an academic research medical center in Boston, Massachusetts which includes Boston University School of Medicine (BUSM) and Boston Medical Center (BMC). BMC is the largest safety-net hospital in New England. Greater than 50% of BMC patients come from underserved populations that depend on government coverage for health expenses through programs like Medicare, Medicaid, and the Health Safety Net [26]. In 2009, 34.4% of the population treated at BMC was White, 31.5% was Black and 17.6% was Hispanic/Latino [27].

The BUMC and BUSM Institutional Review Board approved the protocol. Using electronic medical records (EMR) data, we identified patients who attended outpatient clinic visits as described by Cheng et al. [28]. Briefly, all pelvic ultrasounds from October 1, 2003 through December 12, 2016 were retrieved from the BMC Clinical Data Warehouse (CDW) for women of reproductive age (i.e., between 18 and 45 years old), excluding women with a previous diagnosis of endocrinopathy noted by the following ICD-9 codes and descriptions: 182.0 Malignant neoplasm of corpus uteri, except isthmus; 240.0 Simple Goiter; 240.9 Goiter unspecified; 241.0 Nontoxic uninodular goiter; 241.1 Nontoxic multinodular goiter; 242 Thyrotoxicosis with or without goiter; 243 Congenital hypothyroidism, 244 Acquired hypothyroidism; 245 Thyroiditis; 246 Other disorders of thyroid; 255.0 Cushings Syndrome; 255.1 Hyperaldosteronism; 255.2 Adrenogenital disorders; 255.3 Other corticoadrenal overactivity; 255.4 Corticoadrenal insufficiency; 255.5 Other adrenal hypofunction; 255.6 Medulloadrenal hyperfunction; 255.8 Other specified disorders of adrenal glands; 255.9 Unspecified disorders of adrenal glands; 256.8 Other ovarian dysfunction, in order to determine incidence of PCOM among healthy participants without this previous diagnosis. This process yielded 25,535 unique patient IDs [28]. The time period for data query corresponds to the entire period when ICD-9 coding was in use at BUMC.

### Study design: longitudinal cohort approach

We applied a longitudinal cohort approach using the EMR derived dataset. We identified women undergoing an initial and follow-up transvaginal pelvic ultrasound who received care from 2004–2016 and lived in Massachusetts during this timeframe. Patients were followed through 2016, the last year that air pollution data was available. The first pelvic ultrasound examination over the study period was designated as the initial visit. To establish that women were at risk of PCOM but free of this condition at initial visit, we included only women who had normal ovaries as assessed by the first ultrasound (*n* = 5,492). Follow-up pelvic ultrasound examinations determined the incidence of PCOM.

### Exposure assessment: measurement of fine particulate matter PM_2.5_

We estimated ambient annual average PM_2.5_ using the North American PM_2.5_ model based on the combination of aerosol optical depth (AOD) measurements, the chemical transport model (GEOS-Chem) and geographically weighted regression results, as previously described [29]. Briefly, geophysical PM_2.5_ estimates were consistent with those of globally distributed monitors on the ground (R^2^ = 0.81; slope = 0.90). Geographically weighted regression was also used to account for the residual bias of monitors, producing higher cross validated agreement with ground monitors (R2 = 0.90–0.92; slope = 0.90–0.97) [29]. The PM_2.5_ model yields annual average PM_2.5_ concentration estimates globally at 1 × 1 km resolution, and results are compiled in a freely available database (https://sites.wustl.edu/acag/datasets/surface-pm2-5/#V4.NA.03). These AOD measurements are available at high temporal resolution and provide a historical repository that can be used to retrospectively model PM_2.5_ [30–34]. Annual average PM_2.5_ exposure data starting in the year 2000 were matched to geocoded home addresses from the patient’s initial visit using Esri ArcPro version 2.2 and SAS v. 9.4.

### Outcome assessment: diagnosis of PCOM

We used the novel technique of identifying PCOM or polycystic ovaries based on radiologic report data as described previously using the Rule Based Classifier Model based on the Rotterdam criteria [2, 28]*.* Briefly, an ovary was defined as “PCOM-present” if there were 12 or more 2–9 mm follicles in each ovary and/or if ovarian volume was greater than 10 mL without the presence of confounding pathology [2, 28, 35–37]. Confounding pathology included presence of a dominant follicle (> 10 mm), corpus luteum, abnormal cyst, or ovarian asymmetry, in which case further investigation would be warranted. If a) confounding pathology occurred, b) an ovary was not measured, c) the radiologic ultrasound was not mentioned, or d) PCOM was recorded as absent, we categorized the ultrasound as showing no indication of PCOM and compared this population to patients who had a “PCOM-present” diagnosis.

### Covariates

We extracted EMR information from the patient’s initial visit on demographic characteristics, including age, race/ethnicity, marital status, educational attainment, and smoking status. We calculated body mass index (BMI kg/m^2^) from the height and weight abstracted from this visit. If data on these variables were not available from the initial visit, they were obtained from the visit most proximate to the initial visit within the 2004–2015 timeframe. We restricted our analysis to women with BMIs between 19–54 kg/m^2^ [38], as values outside of this range were not verified and were likely related to documentation errors. Calendar year denoted the year of annual average PM_2.5_ measurement. There were 3,994 women with complete data included in the analysis.

### Statistical analysis

We described the characteristics of the study population using proportions, means and standard deviations. As PM_2.5_ concentrations were measured yearly, we utilized time-varying Cox proportional hazards models to examine the association between PM_2.5_ and the incidence of PCOM. Women contributed person-years starting from January 2000 until ultrasound detected PCOM or the last ultrasound visit. The first pelvic ultrasound examination during the study period confirmed that the patient was free of PCOM at the initial visit. Patients were able to contribute 4 to 15 years of person-time for follow-up. To account for patterns in pollution over time (Figure S1), all models were stratified by age in years and calendar year within the Cox model and were used to estimate hazard ratios (HRs) and 95% confidence intervals (CIs). We categorized air pollution exposure into tertiles in our main analysis to allow for non-linearity and to account for extreme values. The lowest tertile (tertile 1) was designated as the reference group. We conducted multivariate analyses with covariates hypothesized to be associated with air pollution and with PCOM based on a priori literature and directed acyclic graphs [39] (Figure S2). These models included race/ethnicity, educational attainment, marital status, and smoking status [40–44], with educational attainment and marital status serving as proxies for socioeconomic status/household income. We evaluated patients with complete information on all covariates, PM_2.5_ based on complete data on geocoded home address, and PCOM (*n* = 3,994). To evaluate if the association between PM_2.5_ and PCOM varied by BMI and race/ethnicity, we conducted stratified analyses by these variables. As a sensitivity analysis, we also evaluated 1) women who never moved over the study period (*n* = 682) to determine the impact of possible exposure misclassification due to residential mobility and 2) continuous air pollution models to assess precision without categorical restrictions.

## Results

At initial visit, mean age was 31.1 years among the 3,994 women in the analysis (Table 1). The majority of women were Black/African American (57.9%), never smokers (73.5%), and not married (75.2%). About one-third of women graduated high school or received their GED (32.7%) and about one-quarter attained education beyond high school (27.7%). Mean BMI at initial visit was 30 kg/m^3^. Mean PM_2.5_ level from 2004–2016 was 10.0 µg/m^3^, over the entire study period (Table 1).

**Table 1:** Characteristics of Patients at Initial Visit* (2004–2015) **(** ***n*** **= 3994)**

	**% or mean (SD)**
Age (years)	31.1 (7.6)
Race/Ethnicity	
Black/African American	57.9
Hispanic/Latino	5.0
White	15.1
Other	4.8
Declined to Answer	17.2
Educational Attainment	
Some high school or less	37.2
Grad high school/GED	32.7
Some College/Voc/Tech	15.0
Grad college/postgrad	12.7
Declined/Unavailable	2.4
Marital Status	
Married	24.8
Not Married	75.2
Smoking Status	
Current Smoker	19.3
Former Smoker	7.2
Never Smoker	73.5
Mean BMI (kg/m^2^)^a^	30.0 (9.8)
Mean PM_2.5_ (µg/m^3^)^b^	10.0 (1.4)

^*^If not available from initial visit, data obtained from the visit most proximate to initial visit within the 2004–2015 timeframe

^a^% Missing (n): BMI: 4.8% (193)

^b^PM_2.5_ concentration at initial visit

**Fig. 1:**
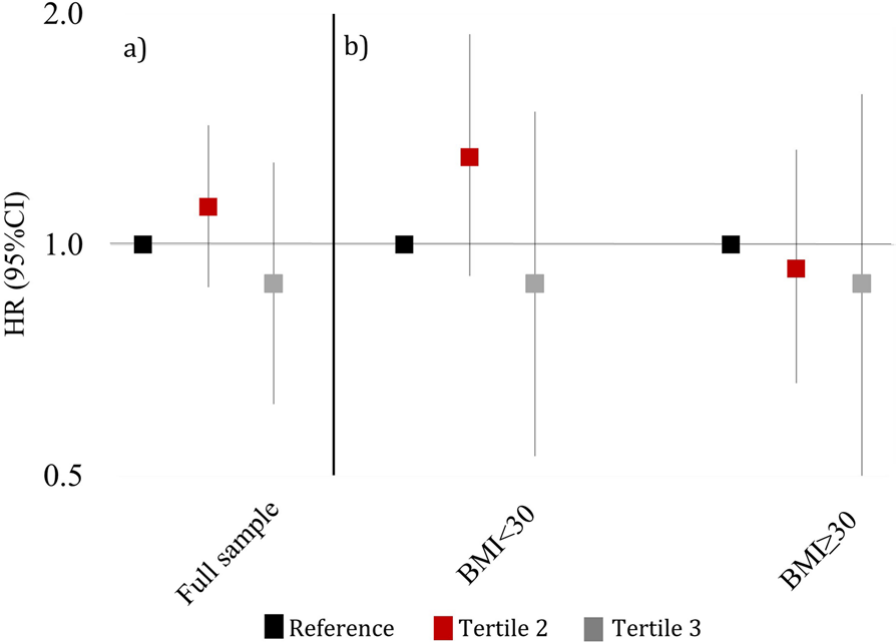
PM_2.5_ and incidence of PCOM for a) full sample (*n* = 3994) & b) BMI (kg/m^2^) stratified analyses (*n* = 3788). Adjusted for race/ethnicity, education, marital status, smoking status; Model stratified by age in years and calendar year

**Fig. 2:**
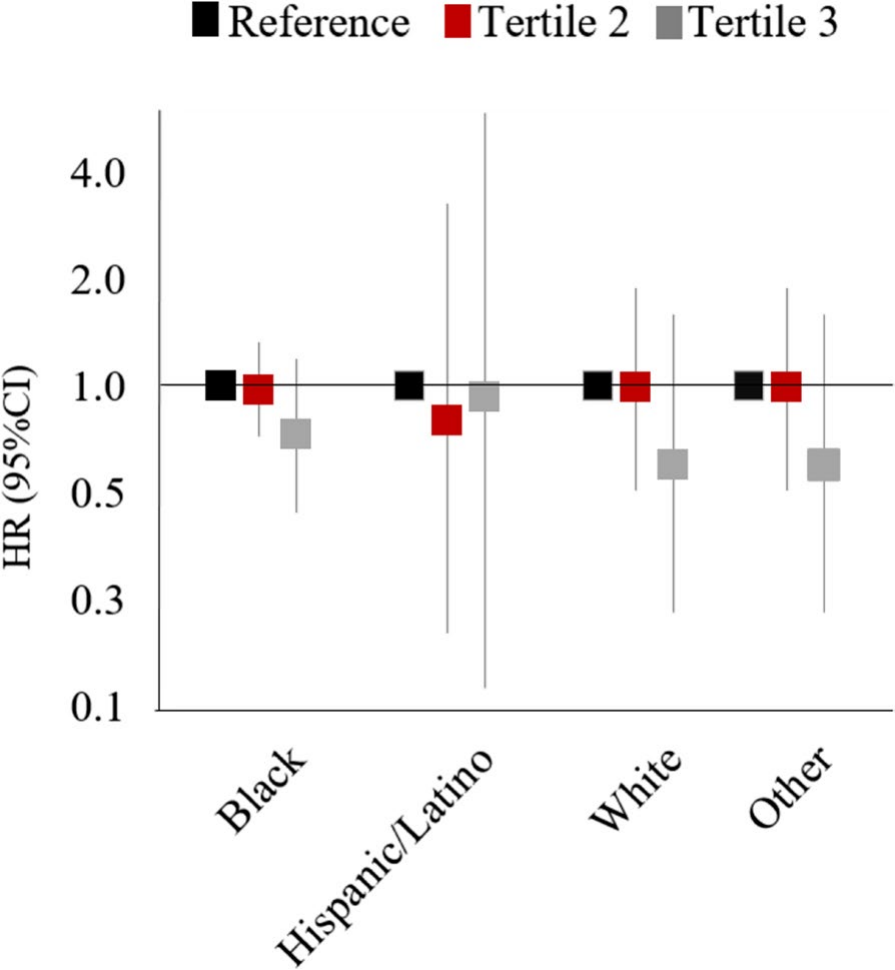
PM2.5 and incidence of PCOM, stratified by race. Adjusted for education, marital status, smoking status. Model stratified by age in years and calendar year. Categories shown for medical record recorded race/ethnicity with highest proportions. Displaying categories for those that self-identified as Black/African America, Hispanic Latino, and White

HRs comparing the second and third tertiles to the reference (first) tertile were 1.12 (95% CI: 0.88, 1.43) and 0.89 (95% CI: 0.62, 1.28), respectively (Table 2, Fig. 1). Thus, we did not observe a dose–response relationships across tertiles. Among women with a BMI < 30 kg/m^2^, HRs comparing the second and third tertiles to the reference tertile were 1.30 (95% CI: 0.91, 1.88) and 0.89 (95% CI: 0.53, 1.49), respectively (Table 3). Among women with BMI ≥ 30 kg/m^2^, we observed weak inverse associations with PCOM for both the second (HR: 0.93, 95% CI: 0.66, 1.33) and third (HR: 0.89, 95% CI: 0.50, 1.57) tertiles when compared to the reference tertile (Table 3, Fig. 1). When stratified by race/ethnicity, the HRs (95% CI) between PM_2.5_ and PCOM among Black, Hispanic/Latino and White women comparing the third tertile to the reference tertile were 0.73 (95% CI: 0.44, 1.20), 0.93 (95% CI: 0.14, 5.90), and 0.60 (95% CI: 0.23, 1.59), respectively (Table 4, Fig. 2). HRs in our sensitivity analysis, restricted to women who never moved, were similar to those for the entire cohort: HRs comparing the second and third tertiles to the reference tertile were 1.25 (95% CI: 0.68, 2.28) and 0.84 (95% CI: 0.33, 2.15), respectively (Table S1). HRs modeling continuous air pollution were null (Table S2).

**Table 2:** Association of fine particulate matter (PM_2.5_) (in exposure tertiles) and Polycystic Ovarian Morphology (*n* = 3994) (complete analysis)

	# Cases	HR (95% CI)^a^	HR (95% CI)^b^
PM 2.5 (µg/m^3^)			
4.90–9.70	798	Reference	Reference
9.80–11.30	319	1.13 (0.88, 1.44)	1.12 (0.88, 1.43)
11.40–17.50	70	0.88 (0.61, 1.25)	0.89 (0.62, 1.28)

^a^Basic model: stratified by age in years and calendar year in the Cox model

^b^Additionally adjusted for race, education, marital status, smoking status

**Table 3:** Association of fine particulate matter (PM_2.5_) (in exposure tertiles) and Polycystic Ovarian Morphology, by BMI status (< 30 vs. >  = 30 kg/m^2^)^a^ (*n* = 3788)

		< 30 kg/m^2^ *n* = 2174			> = 30 kg/m^2^ *n* = 1614
	# Cases	HR (95% CI)		# Cases	HR (95% CI)
PM 2.5 (µg/m^3^)					
5.10–9.70	441	Reference	5.0–9.70	330	Reference
9.80–11.30	171	1.30 (0.91, 1.88)	9.80–11.30	134	0.93 (0.66, 1.33)
11.40–14.80	35	0.89 (0.53, 1.49)	11.40–17.50	26	0.89 (0.50, 1.57)

^a^Stratified by age in years and calendar year in the Cox model; Adjusted for race, education, marital status, smoking status

**Table 4:** Association of tertile fine particulate matter (PM_2.5_) exposure and Polycystic Ovarian Morphology, by race/ethnicity^a^

	Black/African American *n* = 2336	Hispanic/Latino *n* = 204	White *n* = 610	Other *n* = 190
PM 2.5 (µg/m^3^)				
4.90–9.70	Reference	Reference	Reference	Reference
9.80–11.30	0.97 (0.72, 1.33)	0.80 (0.20, 3.27)	0.99 (0.51, 1.90)	1.12 (0.87, 1.43)
11.40–17.50	0.73 (0.44, 1.20)	0.93 (0.14, 5.90)	0.60 (0.23, 1.59)	0.83 (0.58, 1.19)

^a^Stratified by age in years and calendar year; Adjusted for education, marital status, smoking status;

Displaying categories for those that self-identified as Black/African America, Hispanic Latino, and White or as another race/ethnicity

~ 16.5% of participants declined to answer and were not included in this analysis

## Discussion

In this population of women who attended clinic visits at BMC, long-term PM_2.5_ concentrations were not appreciably associated with incidence of PCOM. We observed associations that were inconsistent in direction across tertiles, with no evidence of a dose response relationship. We also found little variation in estimates across race/ethnicity categories, and slight variations across BMI categories, though estimates were imprecise**.**

Previous studies evaluating the association between air pollution and women’s reproductive health outcomes have been limited. A study of 133 Polish women of reproductive age found that higher concentrations of PM_10_, as measured by municipal-level monitoring data, were associated with luteal phase shortening; however, the study did not observe any effect on follicular phase or overall cycle length [45]. A time-series analysis from northwestern China recorded more than 51,893 outpatient visits for menstrual disorders and found that higher short-term ambient PM_10_ concentrations were associated with more outpatient visits for menstrual disorders, with a stronger effect observed among females aged 18–29 years [46]. Furthermore, a cross-sectional study of 34,832 women from the Nurses’ Health Study II observed an association between average total suspended particles with increased odds of androgen excess irregularity phenotypes and lengthened time to cycle regularity [22]. However, none of these studies investigated PCOM explicitly, nor did they assess exposure to fine particulate matter.

Although there is no previous research on air pollution and PCOM, one prior study by Lin et al. has evaluated the relationship between fine particulate matter and PCOS. This prospective Taiwanese study observed that exposure to PM_2.5_ at the fourth (34.78–67.45 ppb) vs. first quartile (22.49- 27.23 ppb) was associated with a 3.56-fold increased risk of PCOS (95% CI: 3.05–4.15) [25]. While the investigators examined PCOS diagnosed with ICD-9 CM codes, they were able to evaluate PM_2.5_ concentrations one year before diagnosis but did not have a longer follow-up, which may have overlooked part of the relevant exposure window within this population. Furthermore, Lin et al. were not able to evaluate effects at lower levels of exposure that are more common in the United States and other countries (mean and 90^th^ percentile weighted annual average across U.S. trend sites in 2019: 7.7, 9.5 µg/m^3^) [47] or to assess the potential for a threshold effect, given the relatively high PM_2.5_ concentrations in the cohort (mean ± standard deviation daily concentrations of PM_2.5_: 30.9 ± 6.2 µg/m^3^). Our study has been able to fill a gap in the literature by specifically evaluating the association between long-term PM_2.5_ and PCOM more commonly observed at lower levels of exposure.

Limitations of the current study include possible restricted generalizability. Our study was limited to women receiving care at BMC and who had an indication for repeated pelvic ultrasounds. Additionally, our EMR dataset was not designed as a traditional prospective cohort study since EMR and air pollution data were both collected before the start of our investigation. However, we were able to assess those at risk of PCOM by only including women with normal ovaries at the first ultrasound visit and at least one repeated pelvic ultrasound examination thereafter to determine development of PCOM. Furthermore, we were unable to confirm if women received care and/or ultrasound examinations at another facility during the timeframe of this analysis. We therefore may not have been able to precisely assess the time to PCOM diagnosis for these women, if, for instance, diagnosis occurred prior to their subsequent ultrasound at BMC. The number of ultrasounds that women underwent and the time between each ultrasound was also not uniform across women. Since PCOM may not cause acute symptoms that indicate an immediate ultrasound, and as women were not screened for PCOM at regular intervals for detection, women may have contributed person time after PCOM occurred but before PCOM was detected via ultrasound. We additionally did not have information accessible to link PM_2.5_ to address changes over time. Consequently, the participant’s address at the initial visit was used to assess PM_2.5_ concentration. Nevertheless, our findings were comparable to results for the entire analytic sample when we restricted our sample to those that had not moved addresses throughout the study. Furthermore, we could not expand our study further to other pollutants in addition to PM_2.5_ because geocoded data on these other components were not available at the time of our analysis.

For this hospital sourced radiological data, ultrasound assessment was not timed to menstrual cycle day, which was also a limitation in our study as we were not able to account for influence of cycle day on ultrasound imaging [48, 49]. However, this study does not evaluate antral follicle count (AFC) measurements on the basal phase of the menstrual cycle (cycle day 2–4), as it was not designed to evaluate AFC in relation to PCOM [50, 51]. Given the age of the population (mean: 31.1; standard deviation: 7.6), we suspect within person variation to be limited. Additionally, those with PCOM at baseline were excluded to evaluate PCOM incidence. Furthermore, our algorithm for detection of PCOM used text from the radiologic report as a proxy, rather than directly counting follicles from ultrasound images. Methods for determining the presence of PCOM in ultrasound reports demonstrated high sensitivity and specificity, and accuracy of up to 97.6% (95% CI: 96.5, 98.5%) when comparing machine learning text algorithm used for classification of PCOM in pelvic ultrasounds based on the radiographic report compared to the hand-labeled test set [28]. However, misclassification of the outcome may have occurred if some providers did not report the necessary information to characterize PCOM, or if there were discrepancies in ultrasound reading by the technician. We also observed marginal inverse associations among women with obesity (BMI ≥ 30 vs. < 30 kg/m^2^) for both higher level tertiles compared to the reference tertile. Yet, results suggesting a potential reduction in incidence of PCOM among obese women may be due to detection bias, as pelvic examinations may be less sensitive for detecting PCOM among obese women.

Additionally, we defined our detection of PCOM based on Rotterdam criteria. Recently, alternative criteria have been proposed including a higher follicle threshold (≥ 25 follicles per ovary), but the sensitivity of these criteria is still being considered [50, 52]. To add further complexity, women with regular menstrual cycles can be defined as having PCOM in the setting of very robust ovarian reserve or younger age [53]. A previous study found the prevalence of polycystic ovaries assessed by antral follicle count to be 32% and that prevalence decreased with age [53]. Future studies should focus on the dynamic aspects of ovarian physiology in an unselected population and include measures of ovarian volume, follicle counts in the basal phase of the menstrual cycle corresponding to follicular recruitment, and include cycle length in the analysis, rather than focusing of PCOM alone.

Although the EMR dataset did not permit us to conduct a traditional prospective cohort study, a strength of our analysis was that we were able to assess those at risk of PCOM over time by having access to baseline and follow-up data. We included women with normal ovaries at the first ultrasound visit and at least one repeated pelvic ultrasound examination thereafter to determine incidence of PCOM. Furthermore, to our knowledge, this is the first study to evaluate exposure to fine particulate matter in relation PCOM. Additional strengths include the efficiency of this analysis in integrating retrospective air pollution assessment in evaluation of reproductive disease pathophysiology. Furthermore, the rich set of EMR data provided a large sample size of nearly 4,000 women and the ability to control for important confounding variables.

## Conclusions

Among a population of reproductive-age women receiving clinical care within our cohort, PM_2.5_ concentrations were generally not associated with higher risk of PCOM at the fine particulate matter levels within our cohort. No dose response relationships were observed nor were estimates appreciably different across race/ethnicity. Future studies with greater variation in exposure levels and additional data on ovarian physiology in unselected populations would further extend these findings.

## Supplementary Information

**Additional file 1:Table S1.** Association of tertile fine particulate matter (PM2.5) and Polycystic Ovarian Morphology (n= 682) among those participants that never moved. **Table S2.** Association of quartile fine particulate matter (PM2.5) exposure and Polycystic Ovarian Morphology (complete analysis, continuous). **Figure S1.** PM2.5 Cumulative Average by Year, averaged across participants (2003-2016). **Figure S2.** Directed Acyclic Graph used to identify covariates for inclusion in regression models as confounding variables; Note: Figure generated using DAGitty v2.3 (Textor and Hardt 2011). Abbreviations: SES, socioeconomic status; PM2.5, fine particulate matter. 

## Data Availability

The datasets generated and/or analyzed during the current study are not publicly available due to confidentiality agreements and the privacy of individuals within the electronic medical records data.
