# Supplementary material for: Fine particulate matter and polycystic ovarian morphology
Source: Environ Health. 2022 Feb 18;21:26. doi: 10.1186/s12940-022-00835-1 (PMC8855564; doi:10.1186/s12940-022-00835-1)
Supplement: Supplementary file 1 — Additional file 1:Table S1. Association of tertile fine particulate matter (PM2.5) and Polycystic Ovarian Morphology (n= 682) among those participants that never moved. Table S2. Association of quartile fine particulate matter (PM2.5) exposure and Polycystic Ovarian Morphology (complete analysis, continuous). Figure S1. PM2.5 Cumulative Average by Year, averaged across participants (2003-2016). Figure S2. Directed Acyclic Graph used to identify covariates for inclusion in regression models as confounding variables; Note: Figure generated using DAGitty v2.3 (Textor and Hardt 2011). Abbreviations: SES, socioeconomic status; PM2.5, fine particulate matter. [file 12940_2022_835_MOESM1_ESM.docx]

**Supplemental Material**

**Fine Particulate Matter and Polycystic Ovarian Morphology**

Victoria Fruh^1^, [Jay Jojo](https://www.ncbi.nlm.nih.gov/pubmed/?term=Cheng%20JJ%5BAuthor%5D&cauthor=true&cauthor_uid=31827874)Cheng^2^, Ann Aschengrau^3^, Shruthi Mahalingaiah*^1,4^, Kevin J. Lane*^5^

*Co-senior authors

^1^Department of Environmental Health, Harvard T.H. Chan School of Public Health, Boston, MA, USA

^2^Department of Biostatistics and Medical Informatics, University of Wisconsin, 702 West Johnson Street, Madison, WI, USA

^3^Department of Epidemiology, Boston University School of Public Health, Boston, MA, USA

^4^Massachusetts General Hospital, Obstetrics and Gynecology, 55 Fruit Street
Boston, MA, USA 02114-2696

^5^Department of Environmental Health, Boston University School of Public Health, Boston, MA, USA

Table S1. A**ssociation of tertile fine particulate matter (PM_2.5_) and Polycystic Ovarian Morphology (n= 682) among those participants that never moved**

|  |  | # Cases | HR (95% CI)^b^ |  |
| --- | --- | --- | --- | --- |
| PM 2.5 (µg/m3) |  |  |  |  |
| **5.10-10.0** |  | **103** | Reference |  |
| **10.10-11.4** |  | **58** | **1.25 (0.68, 2.28)** |  |
| **11.50-17.0** |  | **12** | **0.84 (0.33, 2.15)** |  |
| ^a^Basic model: stratified by age in years and calendar year.  ^b^Additionally adjusted for race, education, marital status, smoking status. | | | | |

Table S2. A**ssociation of quartile fine particulate matter (PM_2.5_) exposure and Polycystic Ovarian Morphology (complete analysis, continuous)**

|  | HR (95% CI)^a^ |
| --- | --- |
|  |  |
| PM 2.5 (µg/m^3^) | 1.002 (0.92, 1.09) |
| IQR PM 2.5 (2.3 µg/m^3^) | 1.004 (0.82, 1.22) |

^a^Stratified by age in years and calendar year in proportional hazards models; Adjusted for race,

exposure years, education, marital status, smoking status

**Figure S1.** PM_2.5_ Cumulative Average by Year, averaged across participants (2003-2016)


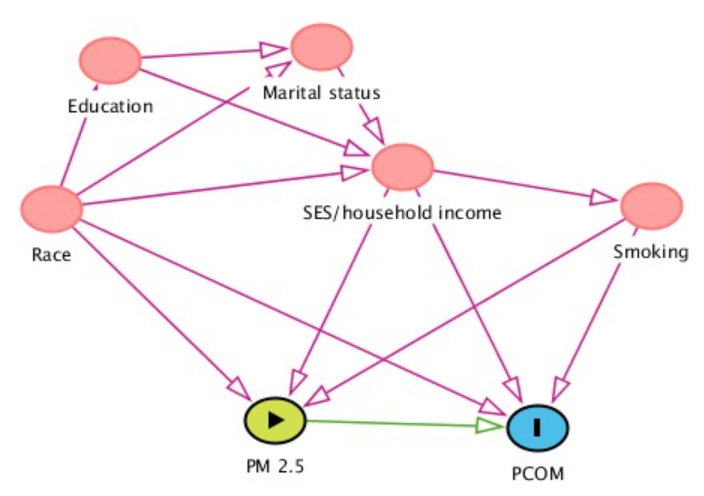


**Figure S2.** Directed Acyclic Graph used to identify covariates for inclusion in regression models as confounding variables; Note: Figure generated using DAGitty v2.3 (Textor and Hardt 2011). Abbreviations: SES, socioeconomic status; PM2.5, fine particulate matter.
